# PWN: enhanced random walk on a warped network for disease target prioritization

**DOI:** 10.1186/s12859-023-05227-x

**Published:** 2023-03-21

**Authors:** Seokjin Han, Jinhee Hong, So Jeong Yun, Hee Jung Koo, Tae Yong Kim

**Affiliations:** 1 Standigm Inc., 70, Nonhyeon-ro 85-gil, Gangnam-gu, Seoul, 06234 Republic of Korea; 2 Standigm UK Co., Ltd, 50-60 Station Road, Cambridge, CB1 2JH UK

**Keywords:** Disease-target identification, Protein–protein interaction, Random walk, Machine learning

## Abstract

**Background:**

Extracting meaningful information from unbiased high-throughput data has been a challenge in diverse areas. Specifically, in the early stages of drug discovery, a considerable amount of data was generated to understand disease biology when identifying disease targets. Several random walk-based approaches have been applied to solve this problem, but they still have limitations. Therefore, we suggest a new method that enhances the effectiveness of high-throughput data analysis with random walks.

**Results:**

We developed a new random walk-based algorithm named prioritization with a warped network (PWN), which employs a warped network to achieve enhanced performance. Network warping is based on both internal and external features: graph curvature and prior knowledge.

**Conclusions:**

We showed that these compositive features synergistically increased the resulting performance when applied to random walk algorithms, which led to PWN consistently achieving the best performance among several other known methods. Furthermore, we performed subsequent experiments to analyze the characteristics of PWN.

**Supplementary Information:**

The online version contains supplementary material available at 10.1186/s12859-023-05227-x.

## Background

Deciphering target proteins for disease treatment has been an important challenge in medical care, as it is the first step in the drug discovery process and one that critically affects its success rate. To effectively solve this problem, we must first understand disease biology, and due to the increased accessibility of high-throughput technologies in recent years, diverse types of unbiased data have been generated for a range of diseases. However, the causes and consequences of disease states are concurrently reflected in those unbiased high-throughput data that compare the disease samples and normal samples. Thus, discriminating potential causes from widespread consequences is an essential task when using disease-perturbed data to prioritize targets to cure.

A well-constructed protein-protein interaction (PPI) network can help tackle this issue because it provides hints for dealing with massive amounts of high-throughput omics data by showing the overall landscape of the protein relations. Several previous studies adopted random walk-based approaches for utilizing PPI networks to associate genes and diseases and reported some encouraging results by suggesting a number of disease genes with literature evidence [1–9]. A biological network consists of nodes and edges, which represent the biological entities and the relations between these entities, respectively. Since the information constituting the network is usually obscured in the given omics data, researchers try to integrate the topological properties into the omics data analysis process and enhance the initial analysis results. One of the methods that leverage the network is a random walk. A random walk diffuses the initial signal through its neighbors. Therefore, the diffused signal is heavily affected by its original signal and the signals derived from the direct neighbors, while other nodes also slightly affect the signal. Most of these studies provided a collective set of known disease genes to initiate random walk processes and obtain novel targets that reflected previously studied disease biology. In the same way, this approach can be applied to extract important genes from omics data. For example, differentially expressed genes derived from omics data can be used as the starting points of random walk processes [10–12].

Most random walk-based methods heavily rely on an unweighted and undirected network when they spread the information assigned to nodes; i.e., they do not make a distinction between different neighbors when choosing which neighbor to use to spread information, although the neighbors have different biological importance levels. Therefore, one can expect that using a *weighted* and *directed* network to propagate more information through important edges can yield improved accuracy. Nevertheless, how to assign suitable edge properties remains a question. In the case of a PPI network, the network contains not just simple interactions between its constituents but much more information, such as the intraconnectivity of protein complexes or a set of proteins involved in the same pathway. This property obviously implies that we must carefully landscape the PPI network to let information flow in the proper direction.

The most intuitive and direct way to achieve this is to use biological information related to the way the researcher wants to. Hristov [11] seems to be a representative example of utilizing this idea. In that study, they showed that the accuracy of random walk algorithm can be improved by using proper prior knowledge. Noteworthy finding among their experiments was that using cancer-specific prior knowledge gives more accurate result than using general cancer-related prior knowledge, in the target identification per cancer type experiment.

Another available option is using the network’s own properties. We choose the curvature in a PPI network, which is based on the local connectivity and other network-derived properties. Curvature is a concept originating from differential geometry that measures the rate of bending at a given point or how much the region in question is *warped* from flat lines, flat surfaces, or flat manifolds. For instance, the curvature of a circle of radius $r$ is $r^{-1}$. Various types of curvatures have been suggested and studied: Gaussian curvature, geodesic curvature, and sectional curvature, to name a few [13]. A Ricci curvature, one of the most important variants, has been used in a wide range of fields, such as fluid mechanics, Einstein’s general theory of relativity, and Perelman’s proof of the Poincaré theorem [14].

We thereby suggest a new algorithm named prioritization with a warped network (PWN), which can be applied to disease target identification methods using a series of high-throughput data, “-omics” data, based on a PPI network. PWN incorporates both network-dependent features and network-independent features to warp the network by applying graph curvature and known disease genes, respectively.

## Results

### Overview of PWN

**Fig. 1:**
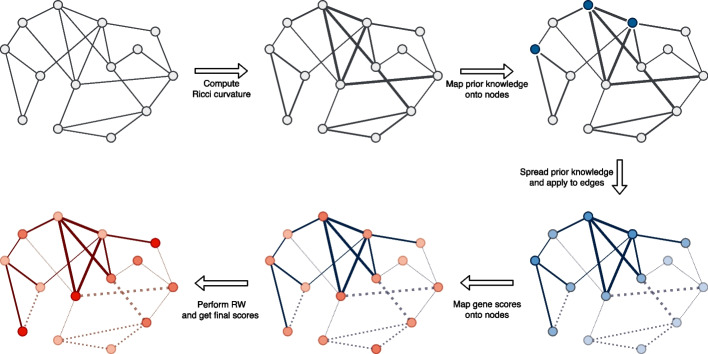
Graphical overview of PWN. PWN generates a weighted and implicitly directed network (lower right) from an unweighted and undirected network (upper left) using two distinct sources. First, PWN computes the Ricci curvature of the edges and derives the first edge weights by applying an exponential function on the computed curvature (upper middle). We consider this curvature an internal feature. After that, the external feature warps the network; prior knowledge is mapped on the network nodes (upper right) and then spread. The spread prior knowledge is then applied to the edges (lower right). Finally, gene scores acquired from the omics data is mapped on the nodes (lower middle) and spread to obtain the final scores (lower left)

PWN is designed to be an efficient variant of random walk with restart (RWR) [15]. Unlike the usual RWR algorithm that employing simple unweighted network, PWN uses a weighted asymmetric network that is generated from an unweighted and undirected network. The weights come from two distinct features. One is an *internal* feature that depends on the network topology, and the other is an *external* feature that is independent of the given network (see Fig. 1 for a graphical overview).

PWN uses the graph Ricci curvature, which is highly related to local structural information, as the internal feature (see “Warping via an internal feature: graph curvature” section). While curvature was originally defined on smooth continuous domains, several researchers have extended this concept to discrete objects such as networks. The graph Ricci curvature [16–19] is one of these extensions that was independently developed by [18] and [16]. The Ollivier–Ricci curvature lies in optimal transport theory, while the Forman–Ricci curvature was derived using CW complexes introduced in homotopy theory. These types of curvatures have been applied to several recent graph-based machine learning algorithms [20–22].

**Fig. 2:**
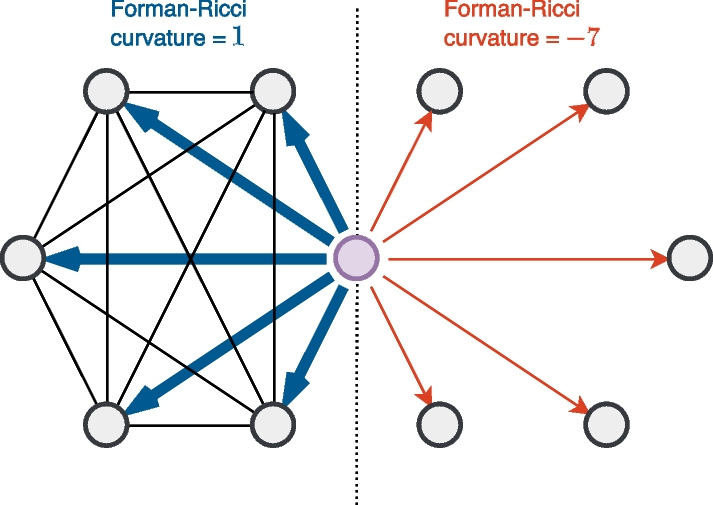
By using curvature, one can precisely control random walks. Consider a random walk starting from the purple node, where one would like to distinguish between the probabilities of being on the left side and right side. Note that the blue edges have curvatures of $1$, while the red edges have curvatures of $-7$, making it easy to control the odds ratio with the curvatures

Note that the edges in a dense complete graph (or clique) tend to have higher curvatures, which implies that higher curvatures can be observed on the edges in intraprotein complexes [23, 24]. In contrast, intercomplex edges have a higher probability of possessing lower curvatures. Therefore, the graph Ricci curvature can be used to overcome the indiscriminate nature of a random walk, as shown in Fig. 2. Consider a random walk starting from the purple node, where the left and right sides have different structures. Naturally, one would like to distinguish them in random walks, but the probabilities of being on the left side and right side are both equal to $5/10=50\%$ when using an unweighted graph. To solve this issue, one can inject the curvature into random walks, and thus, the random walk is now affected by the local structure.

**Fig. 3:**
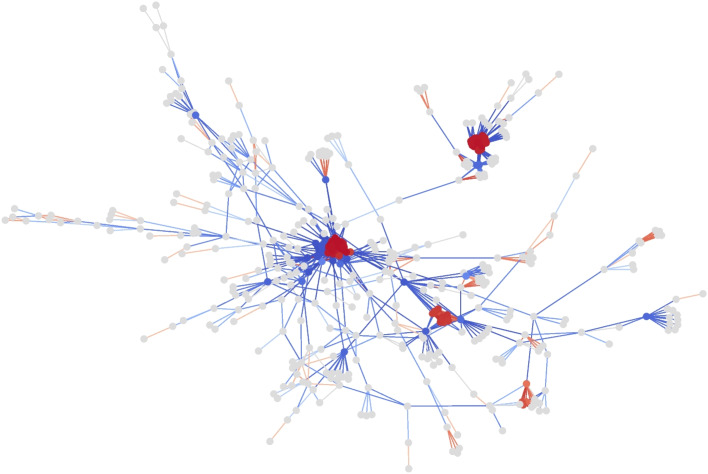
The PPI network of the *E. coli*. The edges are colored using their curvatures, and the nodes are colored using the average of the connected edges’ curvatures. Values close to zero are colored gray, positive values are colored red, and negative values are colored blue. Furthermore, in order to display a more clear difference, nodes with a degree less than 10 were painted gray regardless of the value

In the context of a bioinformatics application, this implies that one can control the amount of information that propagates through or flows out from protein *complexes* and/or *hubs* by using curvature. Proteins are mutually influenced by whether they are physically linked or by various non-physical relationships hidden prior to protein formation (at the level of DNA or RNA). Therefore, it is intuitive that the properties of networks (e.g., hub, curvature, ...) affect the physical or non-physical (information transfer) relationships of protein-protein interaction [25, 26]. Note that most of the nodes belonging to protein complexes are connected to each other, so the edges in the complexes can have large positive curvatures. In contrast, the neighbors of a hub node are likely to not be connected, so the edges attached to the hub have large negative curvatures. It can be seen in various PPI networks that it is possible to distinguish hub nodes from other nodes by a color determined solely by the curvature. Using PPI networks in *E. coli* and humans, we confirmed that these properties are common. As shown in the Fig. 3 and Additional file 1, the protein complexes appear red while the hub proteins appear blue.

In fact, various existing methods already employed Ricci curvatures to their biological applications. Sandhu et al. [22] used curvatures on gene co-expression networks to distinguish between cancer networks and normal networks. Coupled with side information, Sia et al. [27] can construct functional communities with the curvature-based community detection algorithm. Murgas et al. [28] applied PPI curvatures on a single-cell RNA-seq data and obtained successful results on several tasks, which includes distinguishing pluripotent cells and distinguishing cancer cells. Zhu et al. [29] combined the curvature of a PPI with a clustering technique to extract cancer subtypes from the multi-omics data.

PWN is designed to manage the proportion of information circulating in and flowing out of certain regions by controlling this internal feature. We empirically show that this internal feature has little impact on the resulting performance (see “Effectiveness of internal features” section) but provides significant improvements when it is combined with the external feature (see “Effectiveness of the external feature” section).

We use the (augmented) Forman–Ricci curvature instead of the Ollivier–Ricci curvature. Although several studies have suggested that both types of curvature behave similarly on various biological networks (including PPI networks) [30, 31], computing the Forman–Ricci is much faster than computing the Ollivier–Ricci curvature, especially in a large-scale network. Since PPI networks often have large numbers of nodes and edges (see “Collecting PPI networks” section), using the Forman–Ricci curvature seems preferable for us.

After warping the network using curvatures, the prior knowledge (see “Collecting the ground truth and constructing experiments” section) related to the given task is applied to the network as an external feature. While conventional random walk-based methods do not consider the prior knowledge from a given task, some types of modern algorithms, including machine learning methods, heavily employ prior knowledge from the context and encode that knowledge into their algorithms. When attempting to obtain a more appropriate result for a specific task, an algorithm reflecting prior knowledge would perform better than a general task-independent algorithm. Given this assumption, we use external data, which cannot be gathered from the network, to provide a clear guide for the propagation of information and enhance performance.

PWN warps the network by assigning higher weights to prior knowledge-related edges. Note that the prior knowledge is not guaranteed to cover the ground truth in its entirety, so PWN first spreads the prior knowledge, and the missing information can be covered (see “Warping via an external feature: prior knowledge” section).

uKIN, previously suggested by [11], also diffuses the prior information using a (shifted) Laplacian and multiplies the smoothed knowledge with the edge weights. However, uKIN occasionally encounters tuning limitations since the amount of shift is not bounded from above. If the optimal amount of shift is larger than the expected amount, the search space of the tuning hyperparameters rarely contains the optimal region and results in suboptimal performance. Therefore, we use an alternative method based on a RWR [15], where the range of each hyperparameter is always bounded between $0$ and $1$ and is thus easier to tune.

Finally, the gene scores obtained from the unbiased omics data (see “Computing the initial gene scores” section) are diffused through the warped network, which gives the final gene scores (see “Score diffusion with a warped network” section). We add hyperparameters to control the amount of information spread during each step, which makes PWN more versatile and flexible.

### Comparison between PWN and other methods

PWN can be used for identifying the targets with properly given prior knowledge and gene scores. To demonstrate this, we designed series of simulations using various cancer-related data from Homo sapiens, including The Cancer Genome Atlas (TCGA) and Cancer Gene Census (CGC), and check whether the methods can find the known cancer targets.

First, we collect the data as follows (see “Dataset preparation for a simulation study” section for more details). First, we download public PPI databases and compute their initial gene scores using statistical tests performed on transcriptome data. Then, we collect the ground-truth genes and randomly divide them into two groups: one is an (optional) train set used by diffusion methods, and the other is a test set required for performance measurement. From the collected data, we apply various methods and measure the resulting performance metrics. We repeat this multiple times to achieve a robust performance comparison.

Under this setup, we compare the performance of several methods. For baselines, we use the RWR [15], the RWR with GDC [32], uKIN [11], and mND [33], each of which is equipped with the default hyperparameters presented in the original papers. For PWN, we use $\beta =0.5$, $\gamma =0.5$ and $q=0.3$ as the default parameters. Note that these values are not tuned or cherry-picked. Additionally, we add some variant methods to our experiments: the RWR with curvatures, uKIN with curvatures and PWN without curvatures (see “Variants of PWN” section).

**Fig. 4:**
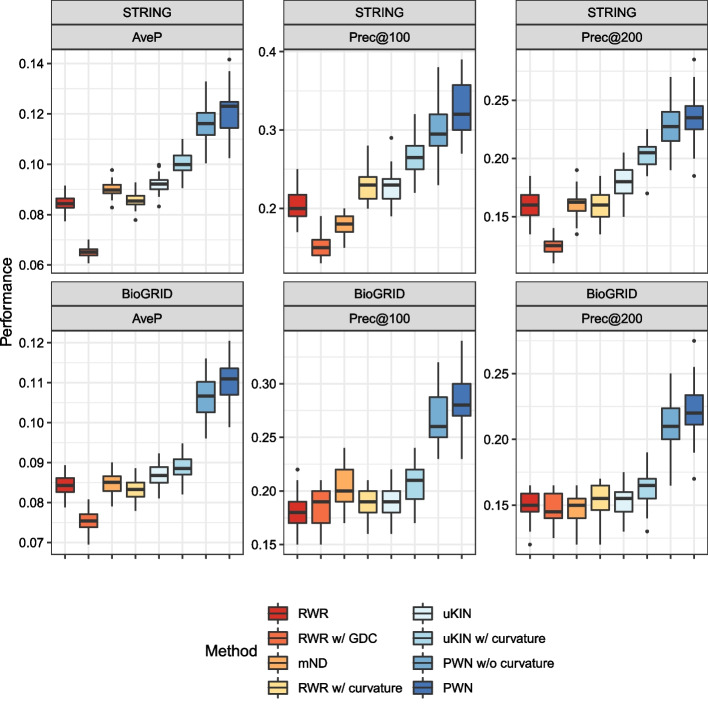
Box plots of the performance metrics. The details are listed in Tables 1 and 2

**Fig. 5:**
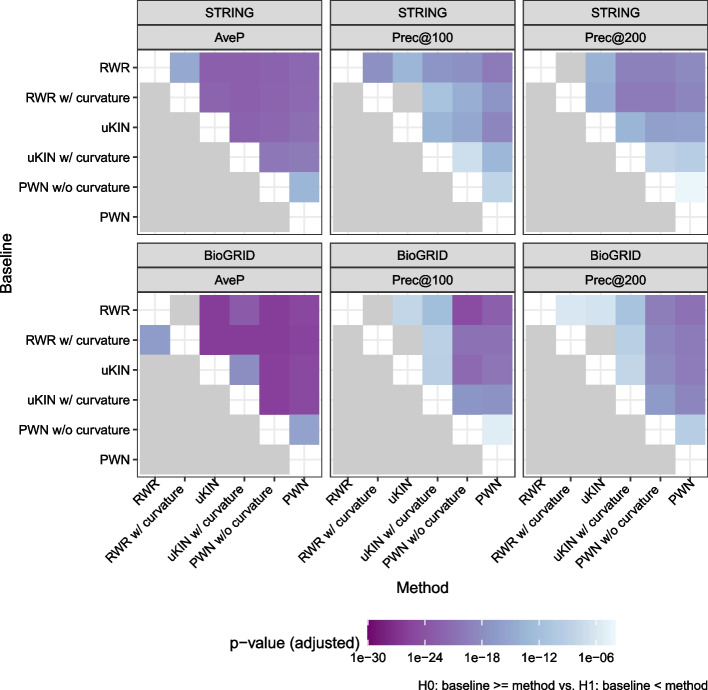
Significance test for performance improvements over baselines. $p$ values are obtained via one-sided paired $t$-tests, and adjusted via the Bonferroni–Hochberg method. Gray color means that the corresponding adjusted $p$ value is larger than $10^{-4}$

We evaluate the performance of each simulated trial and draw box plots for each method and metric, as shown in Fig. 4, Tables 1 and 2, which clearly shows that PWN outperforms the other methods. PWN consistently achieves the highest AveP, with PWN without curvature placing second. Additionally, the significance of the improvement is shown in Fig. 5, which turns out that PWN is significantly better than every other baselines. Also note that the uKIN with curvature performs better than the vanilla uKIN. Contrary to our expectations, the RWR with curvatures does not work well; it is sometimes even inferior to the baseline.

**Table 1:** Detailed performance metrics obtained in experiments using STRING

STRING	AveP	Prec@100	Prec@200
RWR	0.084489 ± 0.002854	0.203000 ± 0.018597	0.160333 ± 0.010822
RWR w/GDC	0.065111 ± 0.001986	0.154667 ± 0.014559	0.123333 ± 0.006989
mND	0.090178 ± 0.003185	0.179000 ± 0.012959	0.160333 ± 0.012658
RWR w/curvature	0.085537 ± 0.003052	0.228000 ± 0.019369	0.158333 ± 0.011321
uKIN	0.092099 ± 0.003555	0.227333 ± 0.020160	0.179500 ± 0.013918
uKIN w/curvature	0.100554 ± 0.004708	0.264333 ± 0.024309	0.204500 ± 0.013980
PWN w/o curvature	*0.116091* ± *0.007404*	*0.299000* ± *0.036041*	*0.225833* ± *0.020556*
PWN	**0.121372** ± **0.008669**	**0.329000** ± **0.036327**	**0.234500** ± **0.022604**

The numbers denote averages and standard deviations. The best performances are in bold; the second-best performances are in italics

**Table 2:** Detailed performance metrics obtained in experiments using BioGRID

BioGRID	AveP	Prec@100	Prec@200
RWR	0.084178 ± 0.002787	0.183000 ± 0.019146	0.149833 ± 0.011332
RWR w/GDC	0.075219 ± 0.002841	0.185333 ± 0.015916	0.147833 ± 0.010803
mND	0.084701 ± 0.002858	0.202333 ± 0.019061	0.148000 ± 0.011641
RWR w/curvature	0.083100 ± 0.002762	0.189333 ± 0.013113	0.153667 ± 0.012172
uKIN	0.086875 ± 0.002995	0.192333 ± 0.016750	0.154667 ± 0.011059
uKIN w/curvature	0.088618 ± 0.003209	0.206333 ± 0.018286	0.163000 ± 0.013235
PWN w/o curvature	*0.106239* ± *0.005078*	*0.265667* ± *0.024870*	*0.209333* ± *0.019728*
PWN	**0.110223** ± **0.005624**	**0.282333** ± **0.026741**	**0.221833** ± **0.021794**

The numbers denote averages and standard deviations. The best performances are in bold; the second-best performances are in italics

In the remaining sections, we focus on analyzing the behavior of PWN, such as the effects of curvature (see “Effectiveness of internal features” section) and prior knowledge (see “Effectiveness of the external feature” and   “Effectiveness of the amount of prior knowledge” sections). Additionally, we find that PWN has slightly more volatile performance than the other methods. The major cause of this variance is identified in “Post hoc analysis of the large induced variance” section.

### Effectiveness of internal features

**Fig. 6:**
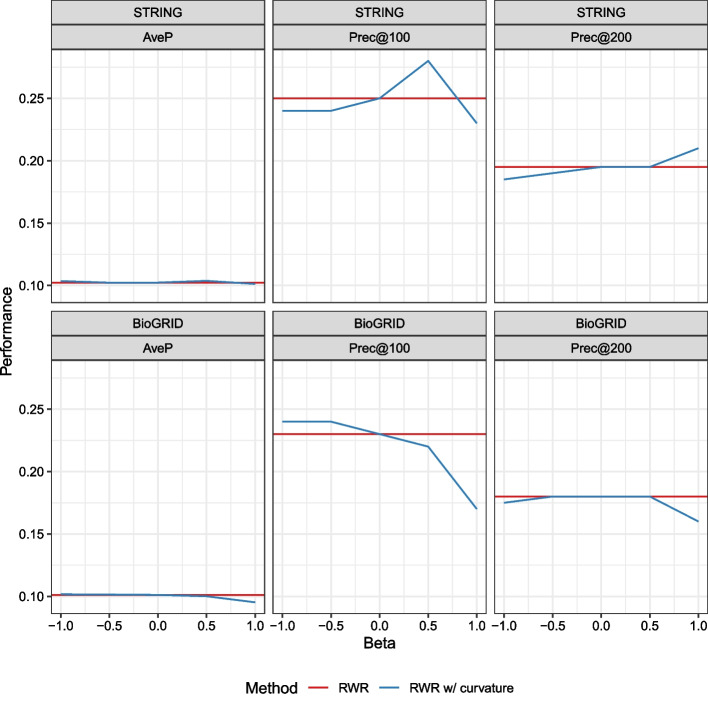
Comparison among methods that do not use prior knowledge

To observe the pure influence of the curvature alone, we compare the methods that do not rely on prior knowledge. Three methods are chosen: the RWR, RWR with GDC [32], and RWR with curvatures. We plot the results in Fig. 6, which reveals that the effect of curvature might be different when using different PPI networks. In the experiment using STRING, one can confirm that Prec@100 and Prec@200 decrease when $\beta$ decreases. In contrast, Prec@100 and Prec@200 decrease when $\beta$ increases if BioGRID is used. Note that AveP seems to remain the same for both cases.

**Fig. 7:**
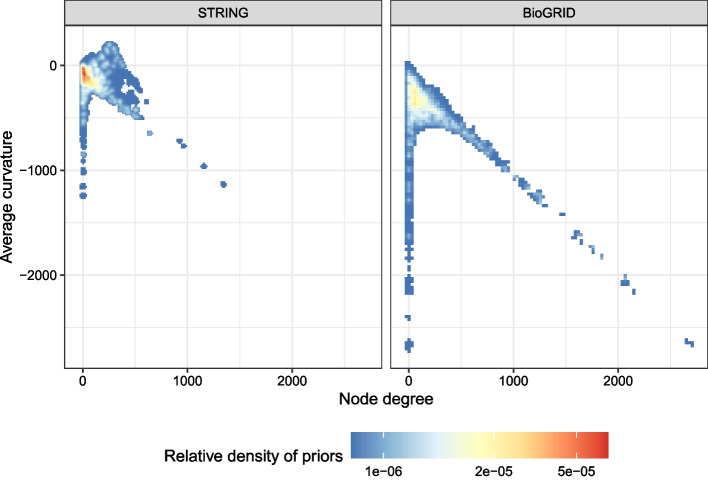
PPI comparison based on average curvatures and node degrees. For each node in the networks, we compute its degree (number of connected edges) and average curvature (mean of the curvatures of its connected edges) and display them on the 2d plane

This phenomenon occurs because the characteristics of the two networks are different, as clearly shown in Fig. 7. Both figures appear to be V-shaped, but some differences are also identified.

- BioGRID has longer tails; in other words, it contains nodes with more extremely negative average curvatures than those in STRING.
- STRING contains nodes with sufficiently positive average curvatures, while BioGRID does not.

From these differences, we suspect that STRING has more protein complexes than BioGRID, while BioGRID tends to have more hub proteins interacting with a large number of neighbors (recall Fig. 2).

Furthermore, notice the difference in perspective of the relative densities of priors. In STRING, the priors are concentrated at near the origin and the positive-curvature region, while the priors are spread more widely and tends to have more negative curvature in BioGRID. These differences seem to have made the difference in Fig. 7; if the priors are in the negative-curvature region (as in BioGRID), it would be advantageous to send prior knowledge towards it by setting $\beta$ to negative and vice versa.

### Effectiveness of the external feature

**Fig. 8:**
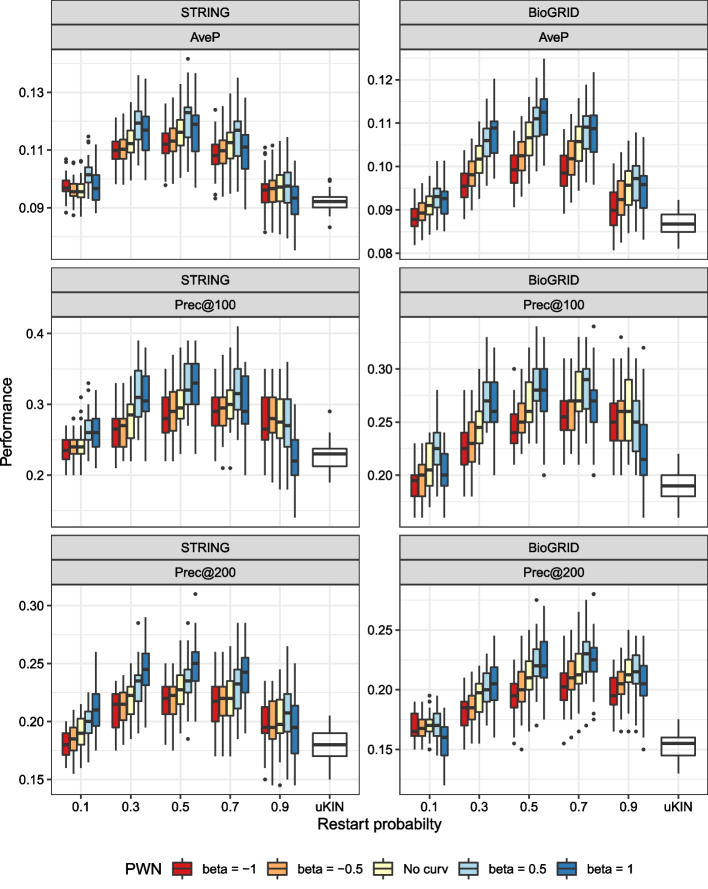
Effect of curvature when curvature and prior knowledge are simultaneously employed. The white boxes are baselines obtained from uKIN

In this experiment, we measure the performance achieved by PWN with varying hyperparameters so that we can understand the comprehensive effect of curvature and prior information. Figure 8 displays the results of an experiment conducted with uKIN as the baseline.

The most remarkable aspect of Fig. 8 is that the application of curvature information enhances performance when used with prior information. PWN with $\beta =1/2$ always performs better than PWN without curvature, as seen when the AveP is calculated. However, larger $\beta$ usually harms the performance again.

**Fig. 9:**
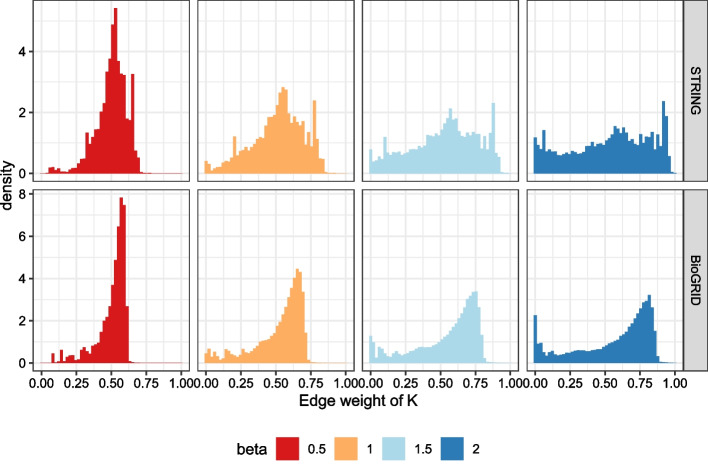
Distributions of edge weights of $K$

We suspect the following hypothesis. Due to the nature of the sigmoid function, as $\beta$ increases, edges weights in the matrix $K$ squashes to 0 s (see Fig. 9). When the edge weight becomes 0, it is impossible for the prior knowledge to be diffused in that direction. Therefore, the prior knowledge does not spread well. Because of this phenomenon, larger $\beta$ interferes the diffusion and harms the performance. Similarly, we suspect that negative $\beta$ also adversely affects the spread of prior knowledge.

Additionally, note that PWN works well when the restart probability $\gamma$ satisfies $0.3\le \gamma \le 0.7$, implying that a moderate level of smoothing is important.

### Effectiveness of the amount of prior knowledge

We have argued that the combination of internal and external features can improve the resulting analysis performance. However, note that external information is not always available. It is important to make PWN work even if there is little prior knowledge; otherwise, PWN could not work on most real-world cases.

**Fig. 10:**
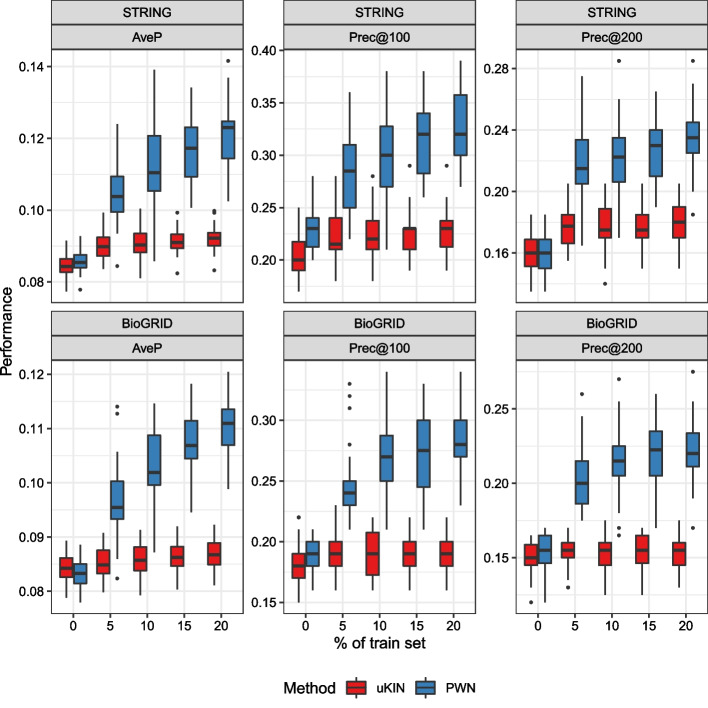
Effect of the amount of prior knowledge

Thus, to determine the influence of the amount of prior knowledge, we plot the performance of PWN and uKIN under various amounts of prior knowledge in Fig. 10. We can observe that PWN always performs better than the baseline as long as prior knowledge is given, regardless of the amount of information. Thus, one can expect a performance improvement even though little prior knowledge is available. In addition, the performance seems to increase linearly as the amount of prior knowledge increases. This implies that PWN can efficiently fuse prior knowledge, since if an information loss exists, the increasing trend is likely to be weak or not detected.

### Post hoc analysis of the large induced variance

**Fig. 11:**
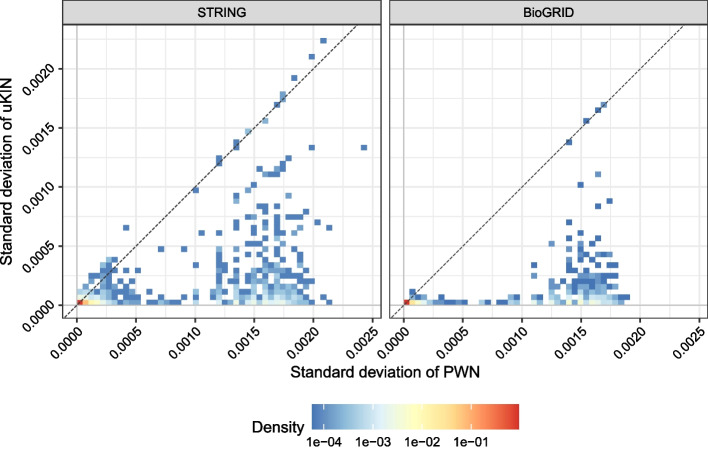
Variance of the smoothed prior knowledge for each gene. The dashed line denotes $y=x$

Although the performance of PWN is superior to that of other methods, we find that the variance of PWN is much larger than that of other methods. We suspect that the large performance variance originates from the large variance of the smoothed prior knowledge, so we empirically verify that hypothesis by comparing the internal variances of uKIN and PWN using the same simulated dataset, and the results are displayed in Fig. 11.

As the figure implies, the standard deviation of the smoothed prior information of PWN is much larger than that of uKIN. We conclude that these high variances of PWN trivially make the transition matrix and resulting gene scores highly volatile, and thus, the performance metrics are affected.

## Discussion

Through the various experiments, we find that PWN performs better than other available random walk methods, although some points related to the effects of using curvatures and the properties of PWN remain to be discovered.

Our first question concerns the reason that a side effect is induced when no prior knowledge is available. We suspect that the curvature itself might have no strong relation to the given task. Topological properties are not explicitly related to biological tasks, and no one can guarantee their effectiveness. Thus, using only topological properties might distort the diffusion process in an unwanted way. Furthermore, due to the massive number of edges, the dissonance becomes large and unrecoverable. As a result, the diffusion process becomes inefficient and might damage the resulting performance. One of our unexpected observations is that applying curvature in a negative sense ($\beta <0$) does not affect or even increases the accuracy.

Utilizing curvatures with prior knowledge, however, has a synergetic effect on the analysis. Note that the amount of prior knowledge is much less than the number of genes in the network; thus, applying prior knowledge removes the effects of most edges by suppressing every edge attached to meaningless nodes. From this observation, we hypothesize that the effectiveness of curvature is at last revealed when the prior information leaves only meaningful and relevant edges; otherwise, the side effect of applying curvature is dominant because there are too many irrelevant edges.

Another noticeable point is related to the effects of the hyperparameters, as shown in Fig. 8. Recall that an extreme value of $\beta$ or $\gamma$ harms the performance. Although we have yet to determine explicit evidence, we interpret this phenomenon as follows. In the case of $\gamma$, we conjecture the following statement: excessive smoothing might cause a slight disparity between the priors and non-priors, and the use of prior knowledge without smoothing can remove important edges related to unobserved or missing knowledge.

Furthermore, a large $\beta$ can make PWN completely ignore low-curvature edges and cause inefficacy in the random walks. As mentioned above, most edges have low edge weights after applying PWN since the prior information suppresses every edge attached to meaningless nodes. Furthermore, since the edges with large curvatures are attached to a few nodes that have many edges, the reconstructed network is very sparse and far away from a single connected network.

### Future works

We’d tested PWN on three PPIs (STRING, BioGRID, IID) and all three experiments confirmed that PWN has the best performance. However, it is a matter to be further confirmed whether this is also true for all PPI. Empirically, it can be verified through other PPI databases or in other organisms.

Default hyperparameters in this paper also have to be verified whether they operate universally well. By our intuition, tuning is necessary depending on the nature of the network. We also think that the larger $\beta$ makes the performance worse, so we can find a range of $\beta$ for the PWN to works well.

Although PWN is very effective, the results of PWN are more volatile than those of other available baselines. We initially suspect that this problem is related to the prior smoothing process due to the following observations: the lack of a variance between PWN and PWN without curvature in Fig. 4, and the intuitive approximated computations (see Additional file 2). Figure 11 empirically proves this hypothesis and provides some hints for reducing the variance. We should build a novel method to effectively smooth the prior information for achieving lower variance.

In this study, we used the driver genes of cancer that have already been experimentally proven as prior information. In addition, in many cases there is significant prior knowledge of the disease, which will affect the performance and application of our algorithms. For example, additional information that can be considered is prior knowledge by cancer type. Interestingly, the more prior knowledge about a particular type of cancer is used, the better we will be able to discover the gene responsible for the cancer. This has already been demonstrated by other groups using similar random walks approach [11].

We tested our idea only on random-work algorithms, but it can be easily extended to other network-based algorithms, especially graph neural networks. Although most graph neural networks are based on message-passing architectures, there exist such cases where random walk is directly used, such as [34], and our idea seems more suitable in the latter case. In line with this, the experiment should be conducted on a task with more complex features and outputs, not just score aggregation.

Lastly, we’re planning to explore other network properties that have greater relevance to disease target identification and employ a high-throughput data analysis to achieve increased performance whether prior knowledge is available or not.

## Conclusion

The random walk approach has become a popular tool in integrative analyses. The trends in recent work suggest that it will continue to be used and further refined as demands related to various data types arise. Several random walk methods have been developed to derive an effective procedure [11, 32]. We introduced PWN, a new method that combines a graph curvature approach for controlling the amount of information flowing in networks with prior knowledge to achieve enhanced prediction performance. We showed above that a synergetic effect was observed when a graph curvature approach and prior knowledge were applied simultaneously. Furthermore, our method achieved the most performance gains relative to GDC [32] and uKIN [11]. In future work, we will also test whether PWN can successfully help analyze other biological data.

## Methods

The Python package and related datasets and code used for our reproducible experiments are available on GitHub.^1^

### Notations

Let $G=(V,E)$ be an unweighted network denoting the interactions between nodes, where $V=\{1,\dots ,n\}$ and $E\subset V\times V$ are sets of nodes and edges, respectively. For $e\in E$, $\sigma _e$ and $\delta _e$ denote the source and target node of edge $e$, respectively, so $\sigma _{(i,j)}=i$ and $\delta _{(i,j)}=j$. Assume that $(i,j)\in E\iff (j,i)\in E$ and $\not \exists i\in V: (i,i)\in E$, which means that $G$ is undirected and has no self-loops. The neighbors of $i$ are defined as $N_{i}=\{k: (i, k)\in E\}$.

Let $A\in {\{0, 1\}}^{n\times n}$ be the adjacency matrix of $G$:

$$\begin{aligned} A_{ji}={\left\{ \begin{array}{ll} 1 &{} \text {if }(i, j)\in E \\ 0 &{} \text {otherwise.} \end{array}\right. } \end{aligned}$$

From the adjacency matrix $A$, the degree matrix $D\in \mathbb {N}^{n\times n}$ is defined as

$$\begin{aligned} D_{ji}={\left\{ \begin{array}{ll} \sum _{k} A_{ki} &{} \text {if }i=j, \\ 0 &{} \text {otherwise.} \end{array}\right. } \end{aligned}$$

### PWN

#### Warping via an internal feature: graph curvature

First, we warp the unweighted adjacency matrix $A$ using the network-related feature. We choose to use the augmented Forman–Ricci curvature [16, 17] $\kappa _{e}$, which can be simply computed as

$$\begin{aligned} \kappa _{e} = 4 - |{N}_{\sigma _{e}}| - |{N}_{\delta _{e}}| + 3|{N}_{\sigma _{e}}\cap {N}_{\delta _{e}}|, \end{aligned}$$

where $|{S}|$ is the number of elements in the set $S$. Then, we construct our first warped adjacency matrix $K=\mathbb {R}_{+}^{n\times n}$ by

$$\begin{aligned} K_{ji} = {\left\{ \begin{array}{ll} \textrm{Sigmoid}\left( \beta \left(\kappa _{(i,j)}-\text{mean}(\kappa)\right)/\textrm{sd}(\kappa )\right) &{} \text {if }A_{ji} = 1,\\ 0 &{} \text {otherwise,} \end{array}\right. } \end{aligned}$$

where $\text{mean}(\kappa)$ and $\textrm{sd}(\kappa )$ are the sample mean and sample standard deviation of $\kappa _{e}$, respectively, and $\beta \in \mathbb {R}$ is a hyperparameter for controlling the effect of curvatures.

Note that the range of $\kappa _{e}$ may differ across various networks and can take extremely large or small values, so we first normalize the curvatures to prevent these potential problems. Additionally, note that $\beta =0$ yields the original unweighted network.

#### Warping via an external feature: prior knowledge

Let the set of prior nodes $P\subset V$ be given, where each node in $P$ is known to be related to a given task and independent of the network. We want to warp $K$ again using $P$ to reflect the prior knowledge. We define $\phi \in \mathbb {R}_{+}^{n}$ as

$$\begin{aligned} \phi _{i} = {\left\{ \begin{array}{ll} 1 / | {P}| &{} \text {if }i\in {P},\\ 0 &{} \text {otherwise.} \end{array}\right. } \end{aligned}$$

Then, we build a Markov kernel $P\in \mathbb {R}_{+}^{n\times n}$ as follows:

$$\begin{aligned} P_{ji} = (1-\gamma )\frac{K_{ji}}{\sum _{k=1}^{n}K_{ki}}+\gamma \phi _{j}, \end{aligned}$$

where $\gamma \in [0,1]$ is a hyperparameter named the restart probability. From the kernel $P$, we compute a stationary distribution $\pi \in \mathbb {R}_{+}^{n}$ and consider it as the smoothed prior knowledge.

$\gamma = 1$ implies the use of prior knowledge without smoothing, while $\gamma = 0$ involves fully smoothing the prior knowledge. Recall that when performing a restart, the kernel jumps to a random prior node that is drawn from some conditionally uniform distribution. This means that the method guarantees the equal use of the prior information.

Finally, we compute the final weighted adjacency matrix $A^{*}\in \mathbb {R}_{+}^{n\times n}$ by

$$\begin{aligned} A^{*}_{ji} = {\left\{ \begin{array}{ll} K_{ji}\pi _{j} &{} \text {if }(i,j)\in {E},\\ 0 &{} \text {otherwise.} \end{array}\right. } \end{aligned}$$

Note that $A^{*}$ is asymmetric, although $A_{ji}^{*}> 0 \iff A_{ij}^{*} > 0$ is still satisfied. In other words, PWN assigns different weights to the same edge but in different directions, which implies that PWN converts undirected graphs to implicitly directed graphs.

#### Score diffusion with a warped network

Let $v^{(0)}\in \mathbb {R}^{n}$ be the initial gene scores obtained from the given omics data. We want to enhance $v^{(0)}$ to $v^{*}$ by injecting more information via the warped network $A^{*}$ so that we can obtain more accurate and reliable scores. We choose to use an RWR as follows:

$$\begin{aligned} v^{(m+1)} = (1-q)A^{*}{D^{*}}^{-1}v^{(m)}+qv^{(0)} \quad \forall m=1,2,\dots ,\qquad v^{*} = \lim _{m\rightarrow \infty }v^{(m)}, \end{aligned}$$

where $q\in [0,1]$ is the restart probability and $D^{*}$ is the degree matrix of $A^*$.

#### Variants of PWN

For qualitative analysis purposes, we also consider the following variants of PWN. The first version is an RWR with curvatures, which considers the $K$ in “Warping via an internal feature: graph curvature” section as a weighted adjacency matrix and applies an RWR on $K$. The second variant is uKIN with curvatures, which applies curvatures to uKIN as in PWN. The last version is PWN without curvatures; i.e., it uses the original adjacency matrix $A$ instead of $K$. Note that this is equivalent to PWN with $\beta =0$.

### Dataset preparation for a simulation study

#### Computing the initial gene scores

We then use unbiased high-throughput data to compute the initial gene scores. The high-throughput data are prepared from TCGA data portal.^2^ We download the transcriptome data containing cancer samples and normal samples for 12 cancer types (breast cancer, colon adenocarcinoma, head-neck squamous cell carcinoma, kidney chromophobe, kidney renal clear cell carcinoma, kidney renal papillary cell carcinoma, liver hepatocellular carcinoma, lung adenocarcinoma, lung squamous cell carcinoma, prostate adenocarcinoma, stomach adenocarcinoma, and thyroid cancer) while considering the balanced availability of the two sample types. Then, we identify differentially expressed genes by computing $p$ values using the $t$-test and combine them using Fisher’s method [35]. In contrast, since mND can handle multidimensional input scores, we do not merge the $p$ values for mND. Finally, we convert the $p$ values to $Z$-scores as $Z=\Phi (1-p)=-\Phi (p)$, where $\Phi$ is the cumulative distribution function of the standard normal distribution.

#### Collecting PPI networks

[36] performed a comprehensive quantitative study comparing the performance and usage of different PPI databases and quantified the agreement between curated interactions shared across 16 major public databases. Among these databases, we exclude 6 databases^3^ that cannot be downloaded and compare the statistics of 10 databases, which are listed in Additional file 3. We select BioGRID [37] and STRING [38] for our main experiments, since BioGRID and STRING provide the largest sets of PPIs among the available primary and secondary databases, respectively. Furthermore, we only include the edges in STRING that have experimental evidence and confidence scores that are larger than 0.7. We also include additional results using IID [39] in Additional file 4.

#### Collecting the ground truth and constructing experiments

For most of the experiments, we need both prior knowledge genes and genes to be uncovered, where the former are used by diffusion methods and the latter are required in performance measurements. We choose to randomly divide the ground truths to simulate this experimental design. If the experiments do not need any prior knowledge (see “Effectiveness of internal features” section), we simply use all ground truths as the genes to be uncovered.

We crawled the CGC list from COSMIC [40] website^4^ as our ground truth. The CGC list contains 723 known cancer driver genes, and we remove 9 genes that are not available in the network. Then, we randomly divide the CGC genes into two subsets at a 2:8 ratio. The smaller train set is employed as prior knowledge for the tested methods, while the larger test set represents the relevant genes to be uncovered by the methods. To achieve robustness, we repeat this process 30 times.

#### Performance measurement

We choose average precision (AveP) and the precision at $k$ (Prec@$k$; $k=100,200$) as the performance metrics, where the definitions are followed:

$$\begin{aligned} \text {AveP}= & {} \frac{\sum _{k} \text {Prec@}k\times \chi (k\text {-th item is relevant})}{\#\text { of total relevant items}},\\ \text {Prec@}k= & {} \frac{\#\text { of relevant items among the top-}k\text { items}}{k}, \end{aligned}$$

where $\chi$ is an indicator function that returns 1 if the given condition is true, and 0 otherwise. Both are commonly used metrics for imbalanced cases in which the portion of the positive class is tiny. Notice that AveP is an estimator for the area under precision-recall curve (AUPRC) [41], and it’s more preferable than directly computing the area since the latter is often known to give overly-optimistic results due to the curve interpolation [42] while computing AveP does not depend on curve interpolation so there is no such problem.

For a fair comparison, we use the exact same prior knowledge and the exact same set of relevant genes for every method in each trial. Additionally, we conduct one-sided paired $t$-tests on the performance metrics to verify that the achieved improvement is significant, and adjust the $p$ values via Benjamini-Hochberg correction [43].

## Supplementary Information

**Additional file 1.** The PPI network of the *Homo sapiens*, colored using the curvatures.

**Additional file 2.** Supplementary information for post hoc analysis purposes.

**Additional file 3.** Summary statistics for the primary/secondary PPIs.

**Additional file 4.** Additional results using IID.

## Data Availability

The software and datasets generated and/or analyzed during the study are available on GitHub: https://github.com/Standigm/PWN.

## Abbreviations

PPI: Protein–protein interaction
RWR: Random walk with restart
AveP: Average precision
AUPRC: Area under precision-recall curve
Prec@$k$: Precision at $k$
TCGA: The Cancer Genome Atlas
CGC: Cancer Gene Census

## Footnotes

1 https://github.com/Standigm/PWN.

2 https://www.cancer.gov/tcga.

3 HitPredict, UniHI, iRefWeb, GPS-Prot, hPRINT and HPRD.

4 https://cancer.sanger.ac.uk/cosmic/census?genome=38.
