# Supplementary material for: PWN: enhanced random walk on a warped network for disease target prioritization
Source: BMC Bioinformatics. 2023 Mar 21;24:105. doi: 10.1186/s12859-023-05227-x (PMC10031933; doi:10.1186/s12859-023-05227-x)
Supplement: Supplementary file 1 — Additional file 1. The PPI network of the Homo sapiens, colored using the curvatures. [file 12859_2023_5227_MOESM1_ESM.pdf]

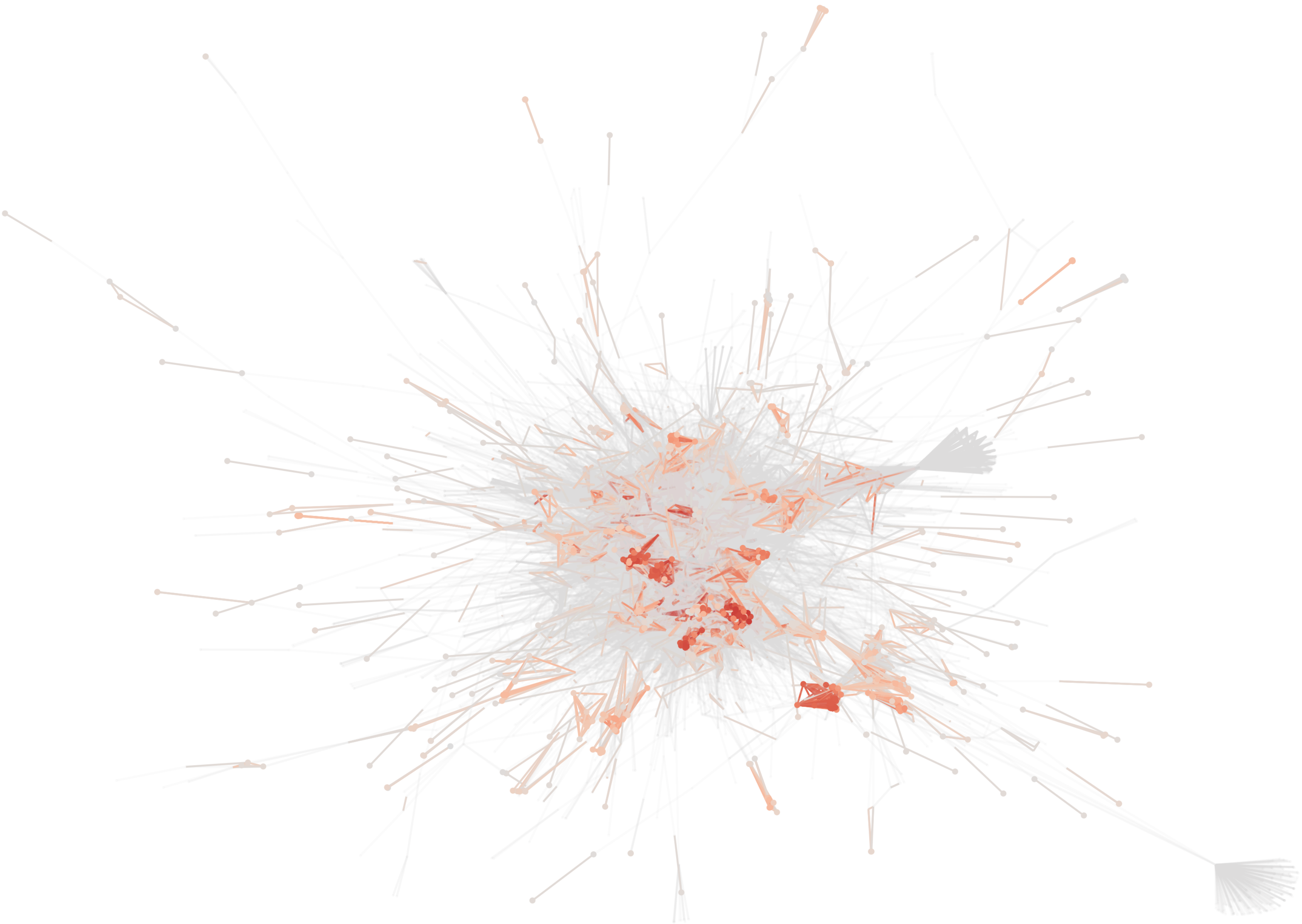

The PPI network of the Homo sapiens.
The edges are colored using their curvatures, and the nodes are colored using the average of the connected edges' curvatures.
Positive values are colored red, while others are colored gray.

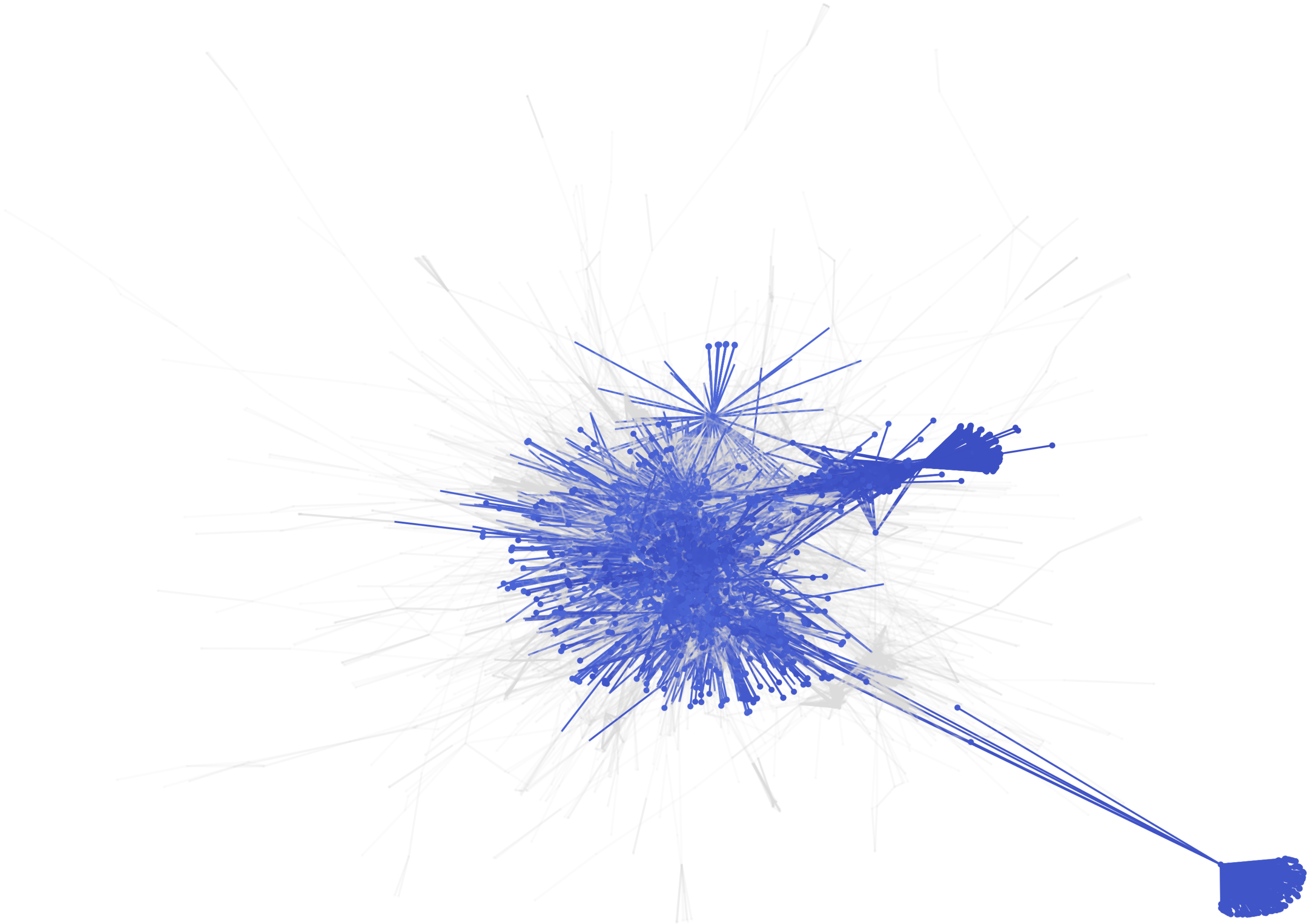

The PPI network of the Homo sapiens.
Values less than -100 are colored blue, while others are colored gray.
