# Supplementary material for: PWN: enhanced random walk on a warped network for disease target prioritization
Source: BMC Bioinformatics. 2023 Mar 21;24:105. doi: 10.1186/s12859-023-05227-x (PMC10031933; doi:10.1186/s12859-023-05227-x)
Supplement: Supplementary file 2 — Additional file 2. Supplementary information for post hoc analysis purposes. [file 12859_2023_5227_MOESM2_ESM.pdf]

1 Supplementary Information for Post Hoc Analysis Purposes

Let $A \in \{0,1\}^{n \times n}$ be the symmetric adjacency matrix of an underlying network, and let $D \in \mathbb{N}^{n \times n}$ be the degree matrix. Then, for any random vector $v \in \mathbb{R}^n$, we define

$$u = (D + \lambda I - A)^{-1} v, \qquad w = (I - \alpha A D^{-1})^{-1} v.$$

Note that u is internally used by uKIN [1], while w is used by PWN to smooth the prior knowledge. Furthermore, assume that $(1-\alpha)D \simeq \alpha\lambda I$. Then,

$$\begin{aligned}
(I - \alpha A D^{-1})^{-1} &= (D - \alpha A)^{-1} D \\
&= [\alpha(D - A) + (1-\alpha)D]^{-1} D \\
&\simeq [\alpha(D - A) + \alpha\lambda I]^{-1} D \\
&= \alpha^{-1}(D + \lambda I - A)^{-1} D.
\end{aligned}$$

Thus,

$$\begin{aligned}
Var(u) &= (D + \lambda I - A)^{-1} Var(v) (D + \lambda I - A)^{-1}, \\
Var(w) &\simeq (I - \alpha A D^{-1})^{-1} Var(v) \left((I - \alpha A D^{-1})^{\top}\right)^{-1} \\
&= \alpha^{-2}(D + \lambda I - A)^{-1} D Var(v) D (D + \lambda I - A)^{-1},
\end{aligned}$$

which implies that $\forall i : \text{sd}(w_i) \propto D_{ii}/\alpha \times \text{sd}(u_i)$. Additionally, note that $D_{ii} \geq 1$, so one can suspect that the variance of w is larger than the variance of u. However, note that this analysis is very rough and does not exactly explain our situation because this assumption is not satisfied in the real scenario.

Bibliography

[1] Borislav H. Hristov, Bernard Chazelle, and Mona Singh. uKIN Combines New and Prior Information with Guided Network Propagation to Accurately Identify Disease Genes. *Cell Systems*, 10(6):470–479.e3, June 2020. ISSN 24054712. doi: 10.1016/j.cels.2020.05.008.
