# Supplementary material for: PWN: enhanced random walk on a warped network for disease target prioritization
Source: BMC Bioinformatics. 2023 Mar 21;24:105. doi: 10.1186/s12859-023-05227-x (PMC10031933; doi:10.1186/s12859-023-05227-x)
Supplement: Supplementary file 4 — Additional file 4. Additional results using IID. [file 12859_2023_5227_MOESM4_ESM.pdf]

1 Additional Results using IID

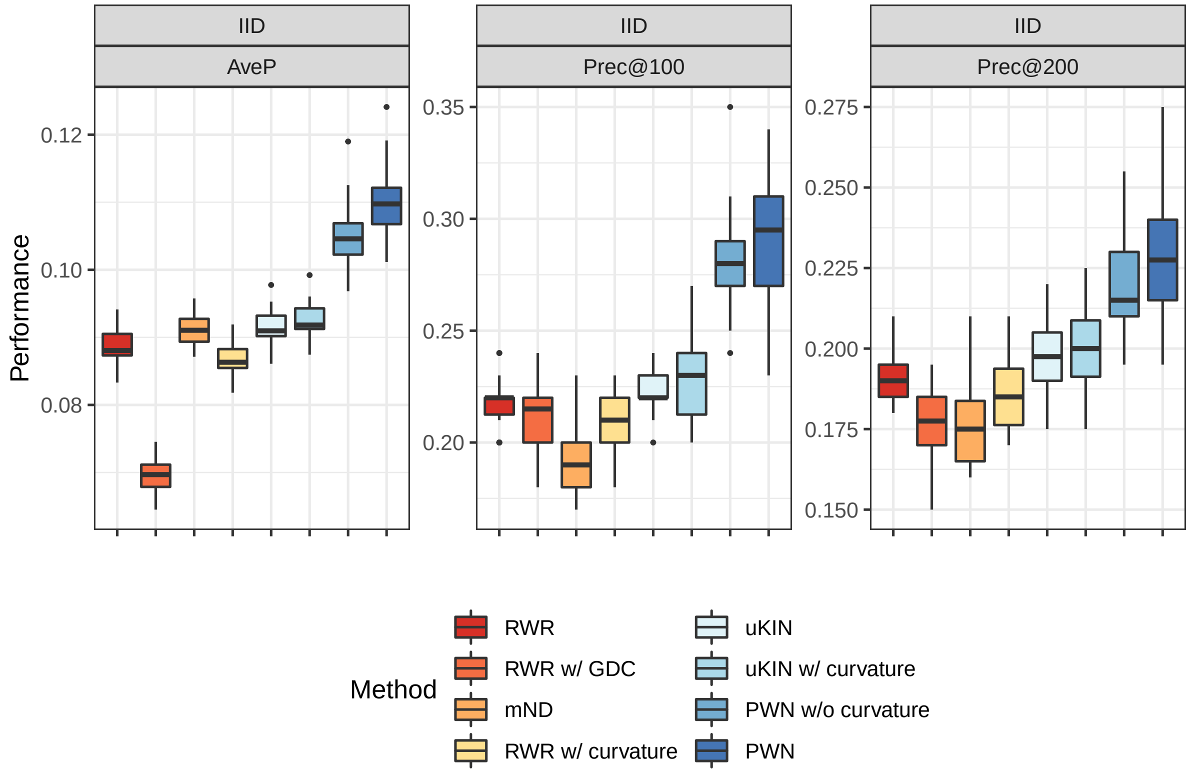

Figure 1: Box plots of the performance metrics. The details are listed in Table 1

Table 1: Detailed performance metrics obtained in experiments using IID. The numbers denote averages and standard deviations. The best performances are in bold; the second-best performances are in italics.

**IID**	**AveP**	**Prec@100**	**Prec@200**
**RWR**	0.088675 ± 0.002431	0.218000 ± 0.009965	0.191667 ± 0.007112
**RWR w/ GDC**	0.069580 ± 0.002418	0.212333 ± 0.013566	0.176833 ± 0.010945
**mND**	0.090937 ± 0.002195	0.193000 ± 0.014420	0.175333 ± 0.011442
**RWR w/ curvature**	0.086769 ± 0.002249	0.208333 ± 0.014875	0.185333 ± 0.009908
**uKIN**	0.091504 ± 0.002551	0.222667 ± 0.010483	0.196167 ± 0.011271
**uKIN w/ curvature**	0.092445 ± 0.002548	0.228667 ± 0.017367	0.199333 ± 0.012507
**PWN w/o curvature**	*0.104957 ± 0.004754*	*0.282667 ± 0.022581*	*0.221167 ± 0.017003*
**PWN**	**0.110032 ± 0.005195**	**0.291333 ± 0.025962**	**0.227333 ± 0.018087**

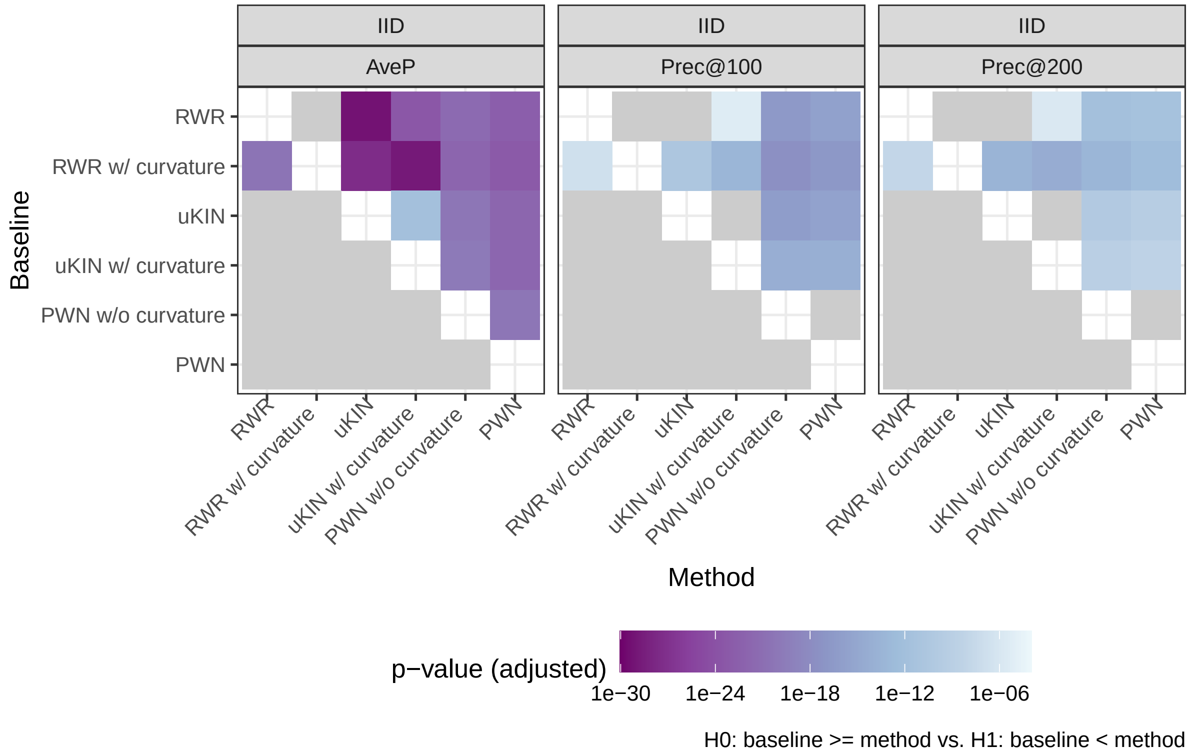

Figure 2: Significance test for performance improvements over baselines. p-values are obtained via one-sided paired t-tests, and adjusted via the Bonferroni-Hochberg method. Gray color means that the corresponding adjusted p-value is larger than 10^{-4}.

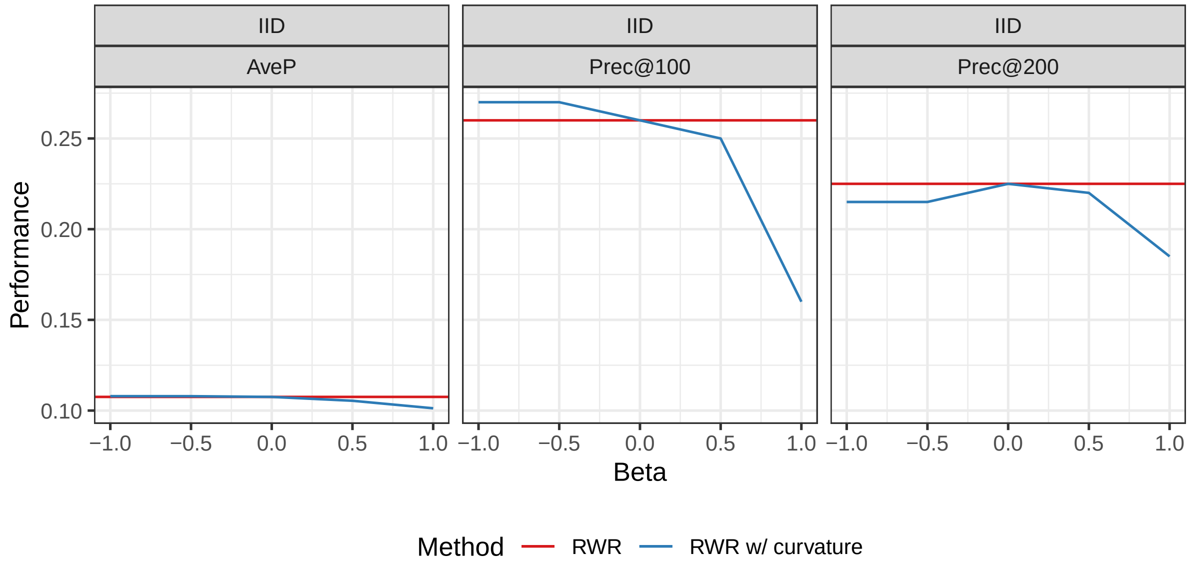

Figure 3: Comparison among methods that do not use prior knowledge.

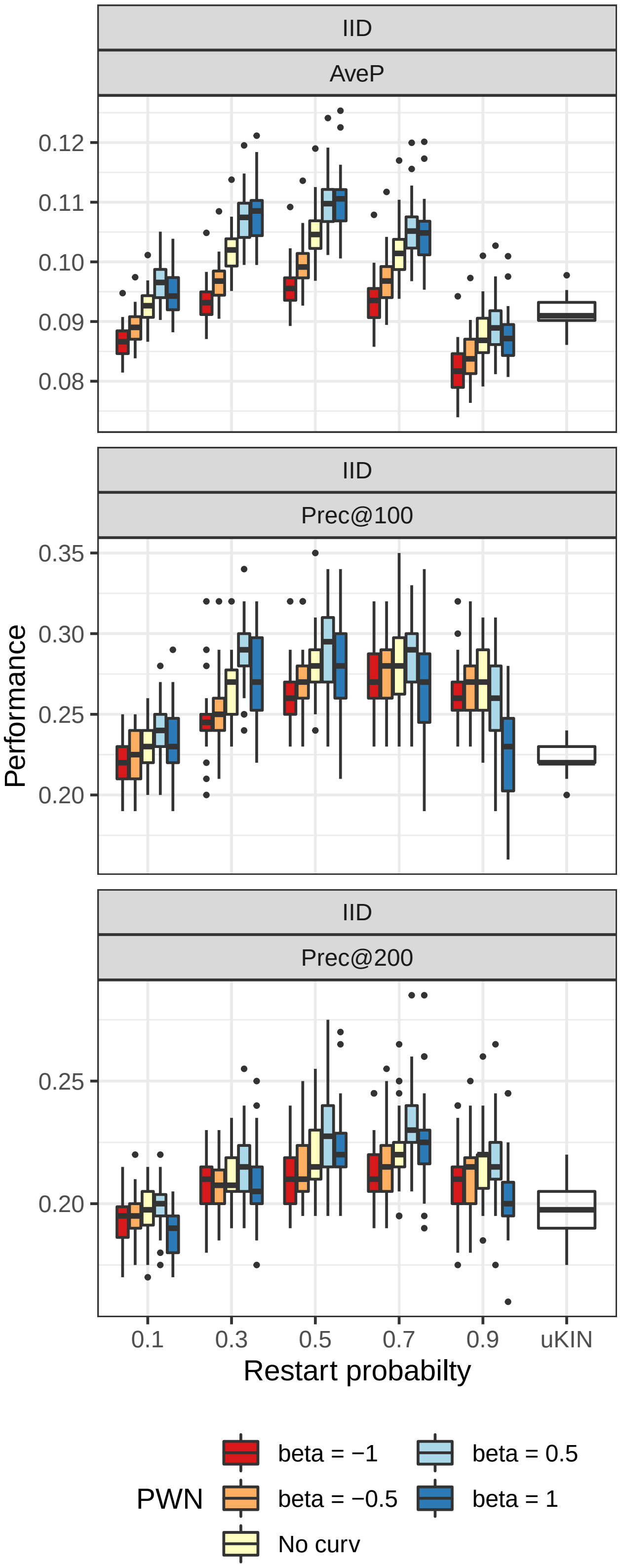

Figure 4: Effect of curvature when curvature and prior knowledge are simultaneously employed. The white boxes are baselines obtained from uKIN.

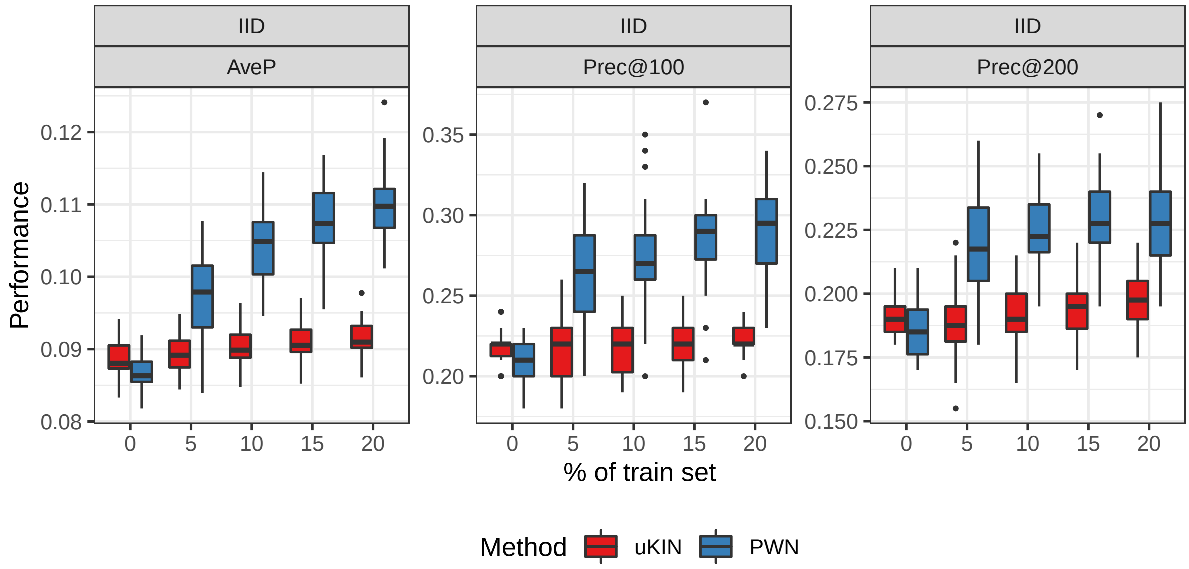

Figure 5: Effect of the amount of prior knowledge.

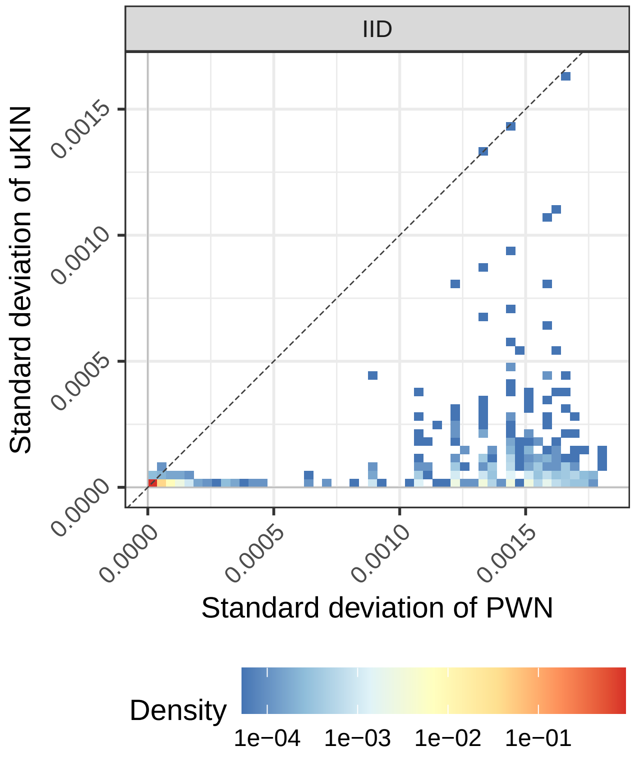

Figure 6: Variance of the smoothed prior knowledge for each gene. The dashed line denotes $y = x$
